# Supplementary material for: Peptidoglycan reshaping by a noncanonical peptidase for helical cell shape in Campylobacter jejuni
Source: Nat Commun. 2020 Jan 23;11:458. doi: 10.1038/s41467-019-13934-4 (PMC6978369; doi:10.1038/s41467-019-13934-4)
Supplement: Supplementary file 1 — Supplementary Information [file 41467_2019_13934_MOESM1_ESM.pdf]

Supplementary Information

Peptidoglycan reshaping by a noncanonical peptidase for helical cell shape in *Campylobacter jejuni*

Kyungjin Min, Doo Ri An, Hye-Jin Yoon, Neha Rana et al.

Supplementary Methods

Pentapeptide synthesis

1) benzyl (((9H-fluoren-9-yl)methoxy)carbonyl)-L-homoserinate

Following a reported procedure, L-homoserine (1.00 g, 8.39 mmol) and $NaHCO_3$ (1.41 g, 16.8 mmol) were dissolved in water (42 mL) at 0 ℃[1]. A solution of Fmoc-OSu (3.12 g, 9.23 mmol) in acetone (42 mL) was added, and the reaction mixture was allowed to stir for 18 h. The reaction mixture was concentrated to give the sodium salt that was dissolved in water (20 mL) and freeze dried. The resulting white solid was dissolved in dry DMF (52 mL) and cooled to 0 ℃. Benzyl bromide (7.18 g, 62 mmol) was added and the reaction mixture was allowed to stir for 3 h at 0 ℃ and then for 12 h at room temperature. The solvent was removed under high vacuo (without heating above 45 ℃) and the resulting residue was taken up in water (70 mL) and extracted with ethyl acetate. The combined organic layers were washed with water (2 × 50 mL), dried ($MgSO_4$) and concentrated in vacuo. The resulting residue was purified by column chromatography to obtain benzyl (((9H-fluoren-9-yl)methoxy)carbonyl)- L-homoserinate (2.64 g, 73%) as a white solid.

2) benzyl (S)-2-((((9H-fluoren-9-yl)methoxy)carbonyl)amino)but-3-enoate

A solution of benzyl (((9H-fluoren-9-yl)methoxy)carbonyl)-L-homoserinate (1.15 g, 2.67 mmol) and 2-nitrophenyl selenocianate (0.72 g, 3.2 mmol) in THF (13 mL) was cooled to 0 ℃ and Bu_3P (0.65 g, 3.2 mmol) was added dropwise in 20 min. The reaction mixture was warmed to 25 ℃ and stirred for 1 h. The reaction mixture was then cooled to 0 ℃, treated with 30% H_2O_2

(1.5 mL) and was kept overnight under stirring at 25 ℃. The resulting mixture was diluted with water (50 mL) and extracted with ether. The combined organic phases were dried (Na and SO) and the solvent evaporated in vacuum. The residue was purified by flash chromatography on silica gel to obtain benzyl (S)-2-((((9H-fluoren-9-yl)methoxy)carbonyl)amino)but-3-enoate (0.46 g, 42%)[2,3].

3) tert-butyl (R)-2-((tert-butoxycarbonyl)amino)pent-4-enoate

N, N'-dicyclocarbodiimide (0.46 g, 2.22 mmol), tert-butanol (0.165 g, 2.22 mmol), and N, N-dimethyl-4-aminopyridine (0.09 g, 0.74 mmol) was dissolved in DCM at 0 ℃. The (R)-2-((tert-butoxycarbonyl) amino) pent-4-enoic acid (0.5 g, 1.48 mmol) was added, and the reaction mixture was stirred at room temperature for 18 h. The reaction mixture was filtered through celite, washed with ether, and filtrate was concentrated by vacuum. Crude compound was washed with water and purified by flash chromatography on silica gel to obtain tert-butyl (R)-2-((tert-butoxycarbonyl)amino)pent-4-enoate (0.53 g, 84%)[4].

4) 1-benzyl 7-(tert-butyl) (2S,6R,E)-2-((((9H-fluoren-9-yl)methoxy)carbonyl)amino)-6-((tert-butoxycarbonyl)amino)hept-3-enedioate

A solution of allyl glycine derivative (0.25 g, 0.91 mmol) and vinyl glycine derivative (0.68 g, 1.64 mmol) in DCM (4 mL) was placed under an argon atmosphere. Grubbs' 2nd generation catalyst (0.039 g, 0.045 mmol) was added and the reaction mixture was stirred at room temperature for 18 h. The mixture was concentrated in vacuo and the residue was purified by flash silica gel column chromatography to obtain 1-benzyl 7-(tert-butyl) (2S, 6R, E)-2-((((9H-

fluoren-9-yl) methoxy) carbonyl) amino)-6-((tert-butoxycarbonyl) amino) hept-3-enedioate (0.38 g, 62%)[3,5].

5) (2S,6R)-2-((((9H-fluoren-9-yl)methoxy)carbonyl)amino)-7-(tert-butoxy)-6-((tert-butoxycarbonyl)amino)-7-oxoheptanoic acid

3% Pt on carbon (0.32 g) was added to a solution of 1-benzyl 7-(tert-butyl) (2S,6R,E)-2-((((9H-fluoren-9-yl)methoxy)carbonyl)amino)-6-((tert-butoxycarbonyl)amino)hept-3-enedioate (0.36 g, 0.54 mmol) in MeOH/H_2O/DCM (9:1:1 v/v/v, 1.1 mL). The reaction mixture was placed under a H_2 atmosphere and stirred for 16 h. On completion of the reaction, the suspension was filtered through a pad of celite, washed with MeOH, and the filtrate was concentrated in vacuo and the residue was purified by flash silica gel column chromatography to obtain (2S, 6R)-2-((((9H-fluoren-9-yl)methoxy)carbonyl)amino)-7-(tert-butoxy)-6-((tert-butoxycarbonyl)amino)-7-oxoheptanoic acid (0.44 g, 95%)[3,5].

6) Penta peptide

The DAP-containing pentapeptide was synthesized under standard Fmoc solid-phase protocol. Wang resin (50 mg, 0.078 mmol, 1.56 mmol g^{-1}) was preswollen with DCM for 30 min and then filtered off. Meanwhile, a solution of Fmoc-D-Ala-OH (5 equiv.), DIC (5 equiv.), HOBt (5 equiv.), and DMAP (0.3 equiv.) in DCM: DMF (2:1, 1 mL) was prepared and stirred for 5 min. The preactivated solution was added to the resin and the reaction mixture was shaken for 3 h. The resin is filtered off and washed with DMF (3 × 5 mL), MeOH (3 × 5 mL) and DCM (3 × 5 mL). The reaction completeness was confirmed by Kaiser Test. After completion of coupling, the Fmoc protecting group was removed with a 20% solution of piperidine in DMF (3 × 10 min)

and then washed with DMF (3 × 5 mL) and DCM (3 × 5 mL). After the deprotection of Fmoc group, the resin was treated a solution of Fmoc-D-Ala-OH (5 equiv.), HBTU (5 equiv.), HOBt (5 equiv.), and DIPEA (10 equiv.) in DMF (1 mL). The reaction mixture was shaken for 90 min. The resin was then filtered and washed with DMF (3 × 5 mL) and DCM (3 × 5 mL). The protocols for Fmoc deprotection and HBTU coupling repeated with Fmoc-D-Glu-OtBu and Fmoc-L-Ala-OH. Afterwards, a solution of Ac_2O (10 equiv.) and DIPEA (10 equiv.) in DMF (1 mL) was added to the Fmoc deprotected resin and then shaken for 80 min. The resin was then filtered and washed with DMF (3 × 5 mL), MeOH (3 × 5 mL) and DCM (3 × 5 mL). The peptide was cleaved from the resin by shaking in a solution of TFA/TIS/H_2O (1 mL, 95:2.5:2.5) for 2 h. The resin was filtered, washed with TFA (7 mL) and the combined filtrate was concentrated in vacuo. The resulting residue was precipitated from cold ether (15 mL) to obtain a white product. The peptide was then centrifuged and the cold ether was carefully removed. The precipitated peptide was dissolved in water (10 mL) and lyophilized to obtain a white powder that was purified by preparative RP-HPLC (4.9 mg, 11% overall yield). MS (ESI) m/z: Anal. calcd. for [M+H] $C_{23}H_{39}N_6O_{11}$: 575.3; found 575.3[3,5].

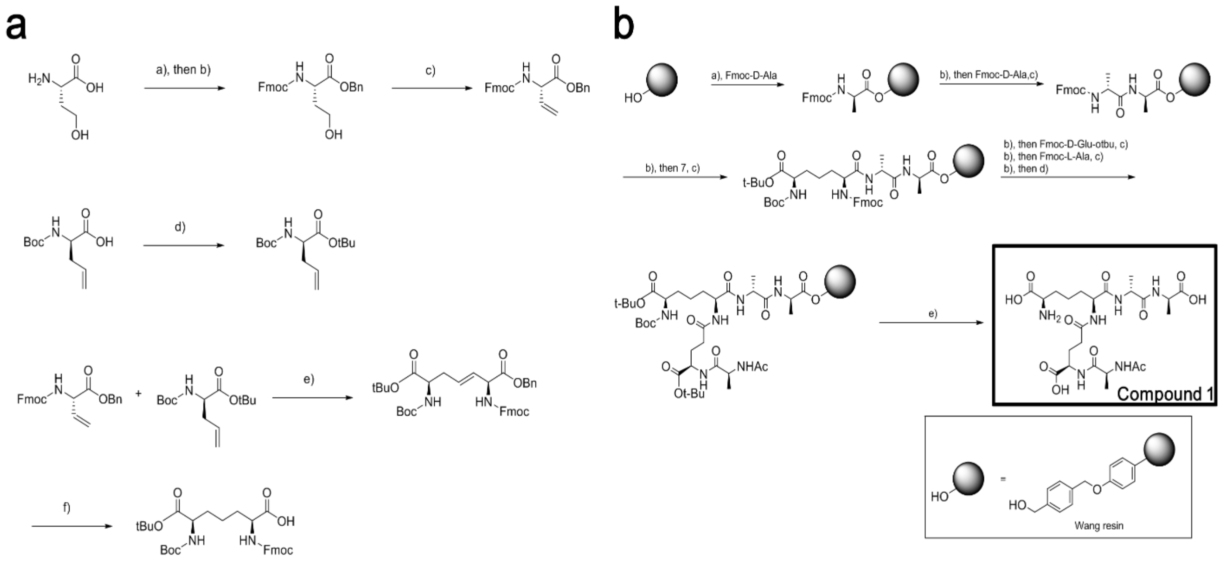

Supplementary Figure 1. Pentapeptide synthesis process. a, Meso-DAP derivative synthesis scheme 1. a) Fmoc-OSu, $NaHCO_3$, Acetone / H_2O, 0 ℃ to RT, 18 h, 90%. b) BnBr, $NaHCO_3$, DMF, 0 ℃ to RT, 15 h, 73%. c) i) $NO_2PhSeCN$, Bu_3P, THF, ii) 30% H_2O_2, 42%. d) t-BuOH, DCC, 4-DMAP, DCM, RT, 20 h, 84%. e) Grubbs 2nd gen cat., DCM, RT, 18 h, 62%. f) 3% Pt/C, H_2 gas, MeOH/H_2O/DCM (9:1:1), 16 h, 95%. **b,** Peptide cross-linked process scheme 2. a) DIC, HOBt, 4-DMAP in DCM/DMF (2:1). b) Piperidine in DMF (20 %). c) HBTU, HOBt, DIPEA in DMF. d) Ac_2O, DIPEA in DMF. e) TFA/TIPS/H_2O (95:2.5:2.5).

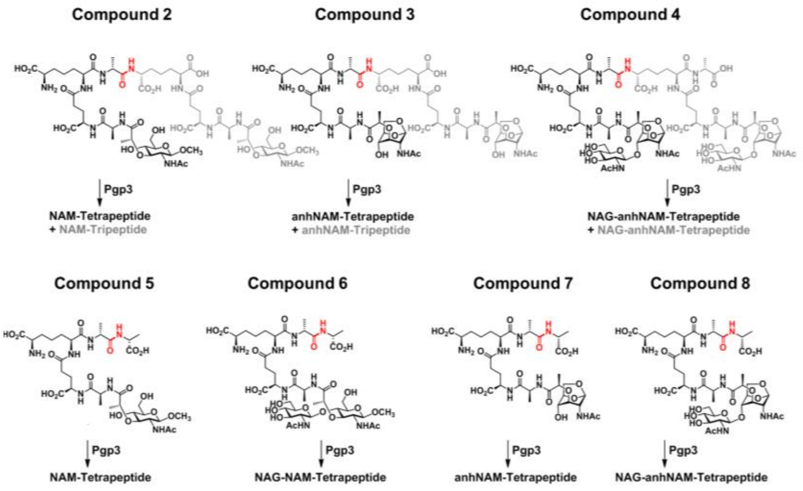

Peptidase activities of Pgp3		
D,D-Endopeptidase activity	2	70%
	3	95%
	4	100%
D,D-Carboxypeptidase activity	5	100%
	6	87%
	7	100%
	8	100%

Supplementary Figure 2. Synthetic peptidoglycan substrates and reaction products. Chemical structures of the synthetic peptidoglycan substrates used in this study and their corresponding Pgp3 reaction product. Percentage of product formation for a given substrate.

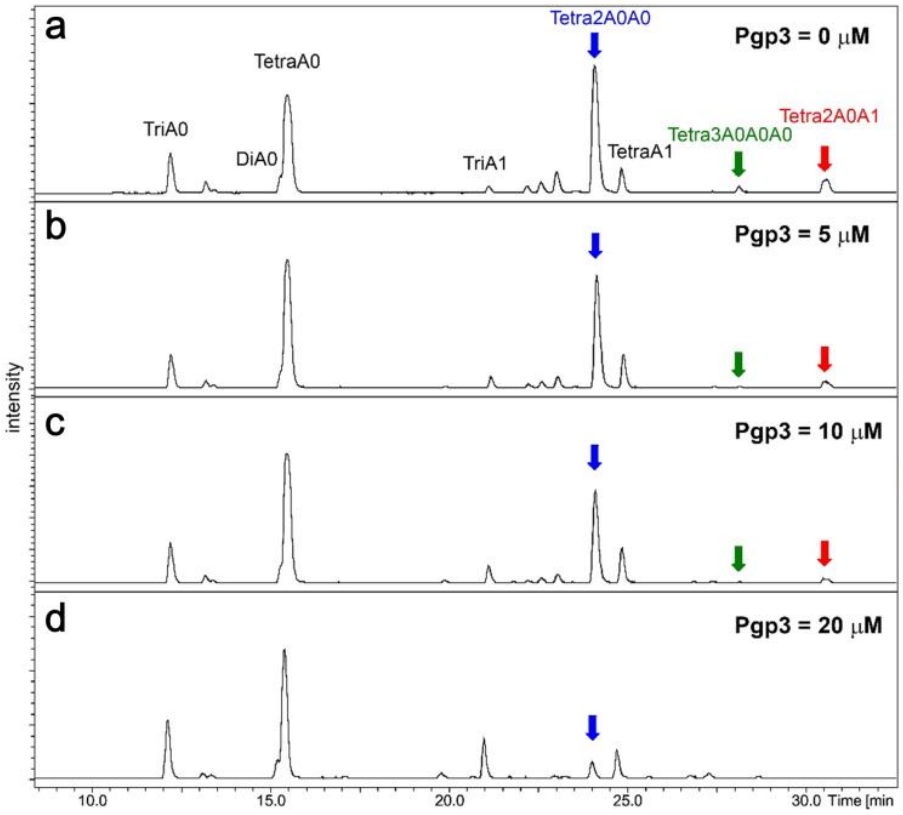

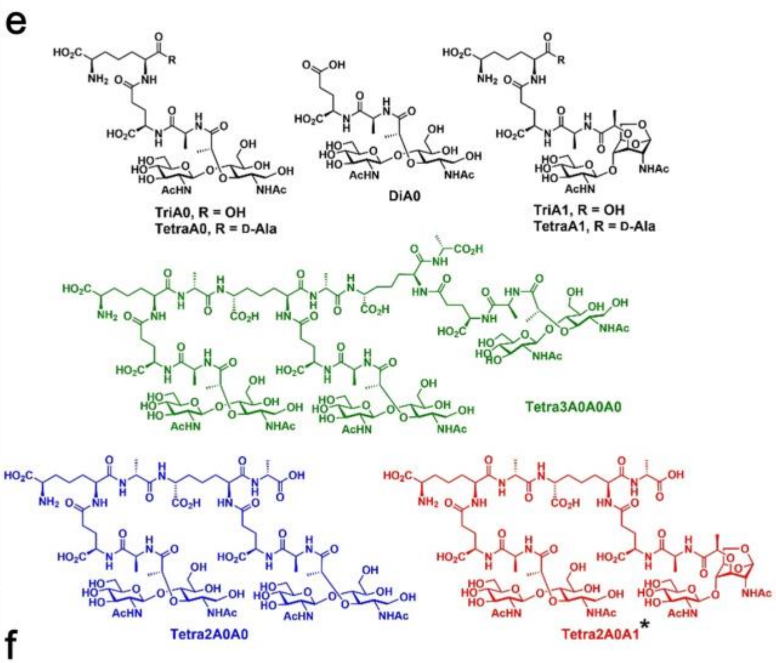

f

Pgp3 [μM]	0	5	10	20
Non-crosslinked	58 (5)	61 (3)	72 (4)	94 (2)
Crosslinked	42 (5)	39 (3)	28 (4)	6 (2)

Supplementary Figure 3. The reaction of sacculus with Pgp3, followed by mutanolysin. a-d, LC-MS traces of the reactions with varying concentration of Pgp3. **e**, Chemical structures of major products from the reactions. **f**, Summary of product analysis of the reactions (amounts are expressed as a percentage of the total extracted-ion chromatogram peak area; average values of three runs with error in parenthesis). *The position of NAG-anhNAM was not determined (panel **e**). The given structure is one of two possible isomers. Colored structures (in blue, green and red) in panel **e** are muropeptides containing D,D-crosslinks, substrates for Pgp3. Each is shown by correspondingly colored arrows. The fate of those substrates is shown in panels **a-d**. As the concentration of the enzyme is increased, less substrate survives.

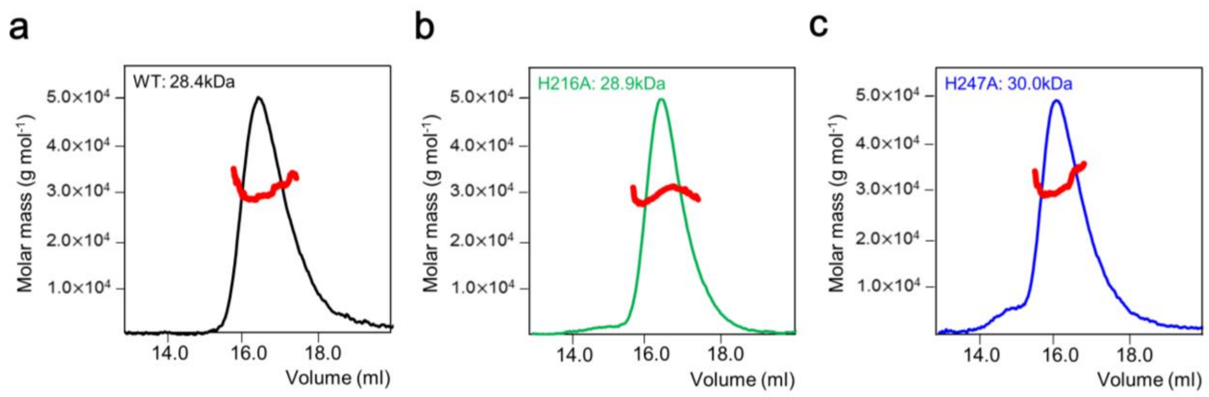

Supplementary Figure 4. SEC-MALS experiment. SEC-MALS profiles for **a,** WT Pgp3 (black), **b,** H216A mutant (green), and **c,** H247A mutant (blue). The thick red lines represent the measured molecular weight.

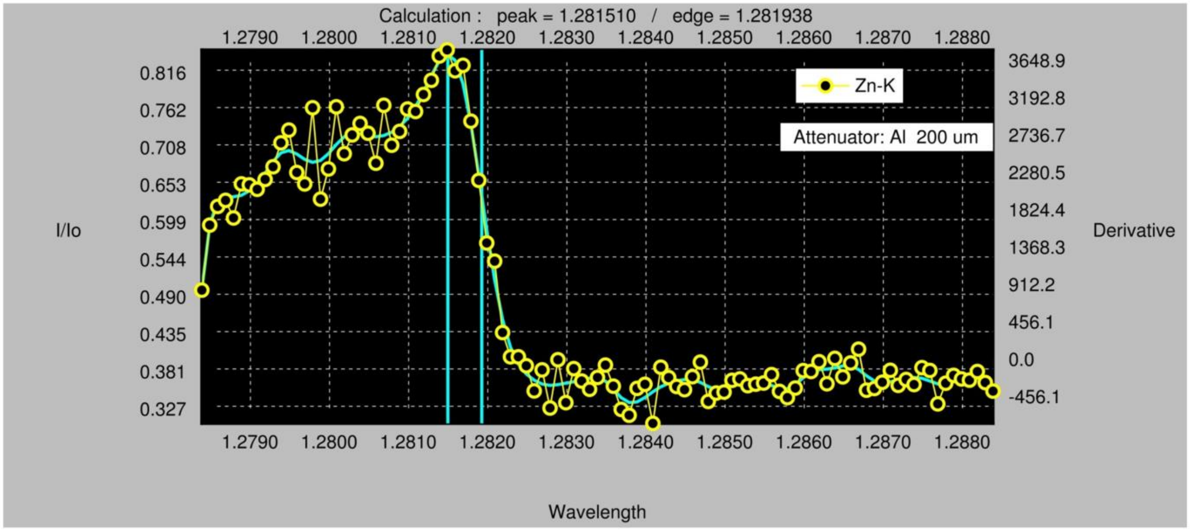

Supplementary Figure 5. X-ray absorption data for Zn^{2+}.

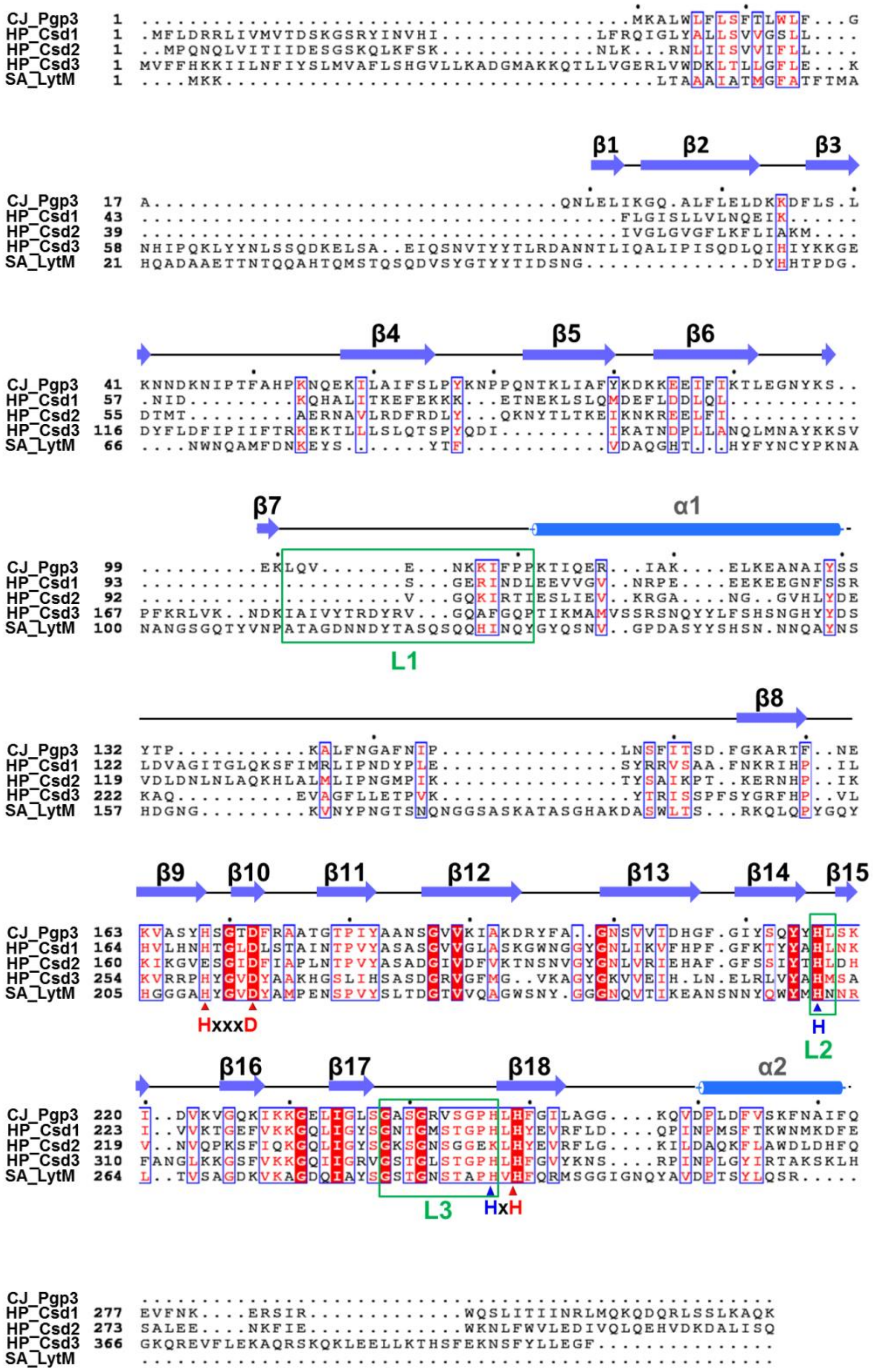

Supplementary Figure 6. Sequence alignment of *C. jejuni* Pgp3 with its homologs. Multi-alignment of Pgp3 (UniProtKB/Swiss-Prot accession number A8118_01115) against Csd1 from

H. pylori (UniProtKB/Swiss-Prot accession number HP1543), Csd2 from *H. pylori* (UniProtKB/Swiss-Prot accession number HP1544), Csd3 from *H. pylori* (UniProtKB/Swiss-Prot accession number HP0506), and LytM from *S. aureus* (UniProtKB/Swiss-Prot accession number O33599). Secondary structural elements were assigned using PyMOL and every tenth residue is marked with a black dot. Red triangles indicate the conserved residues in HxxxD and HxH motifs that are important for the metallopeptidase activity. Three loops are indicated by green boxes. Blue triangles indicate the conserved residues for water coordination in substrate hydrolysis activity.

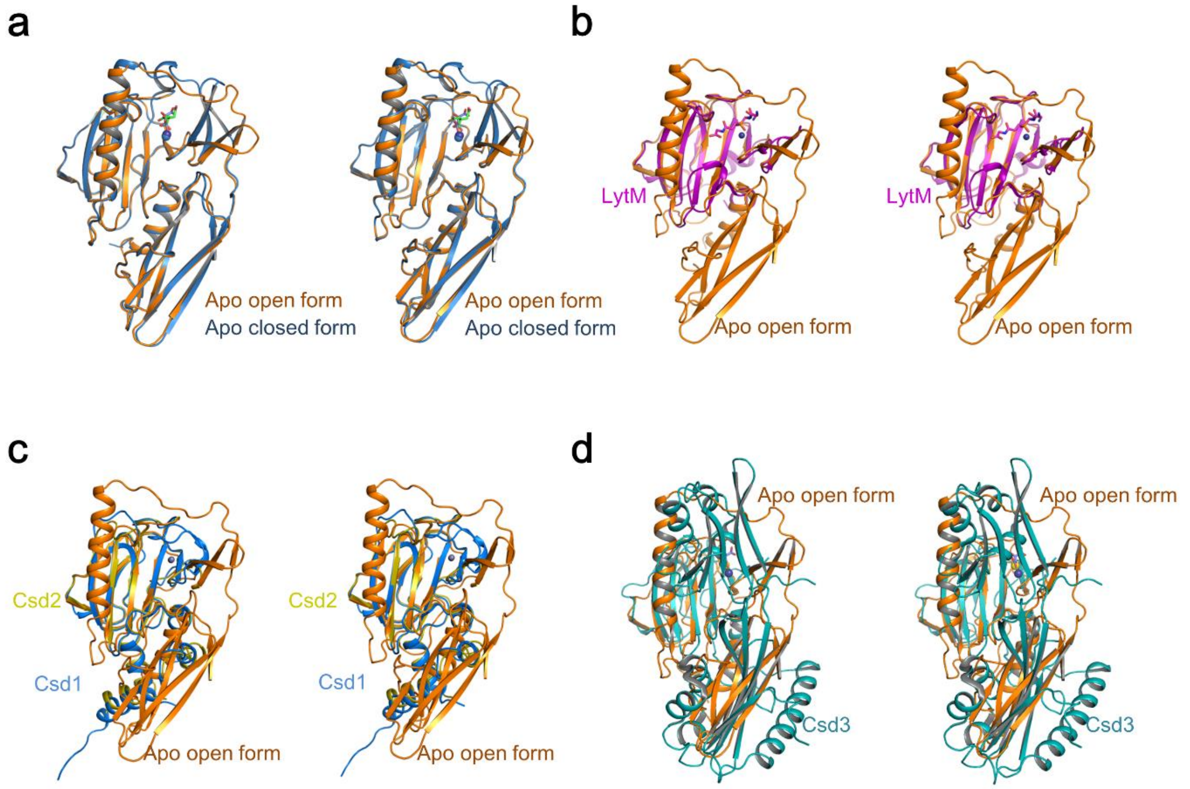

Supplementary Figure 7. Structural comparisons between Pgp3 and M23 metalloprotease. a, Structural comparison between open (orange, PDB ID: 6JMX) and closed forms (blue, PDB ID: 6JMX) of Pgp3. **b,** Stereo view of the superposition between the LytM domain from *S. aureus* (PDB ID: 4ZYB, magenta) and WT Pgp3 (open form, orange, PDB ID: 6JMX). **c,** Stereo view of the superposition between Csd1/Csd2 from *H. pylori* (PDB ID: 5J1L and 5J1K), and WT Pgp3 (open form, PDB ID: 6JMX), shown in blue, yellow, and orange, respectively. **d,** Stereo view of the superposition between Csd3 from *H. pylori* (PDB ID: 4RNZ, dark cyan) and WT Pgp3 (open form, orange, PDB ID: 6JMX).

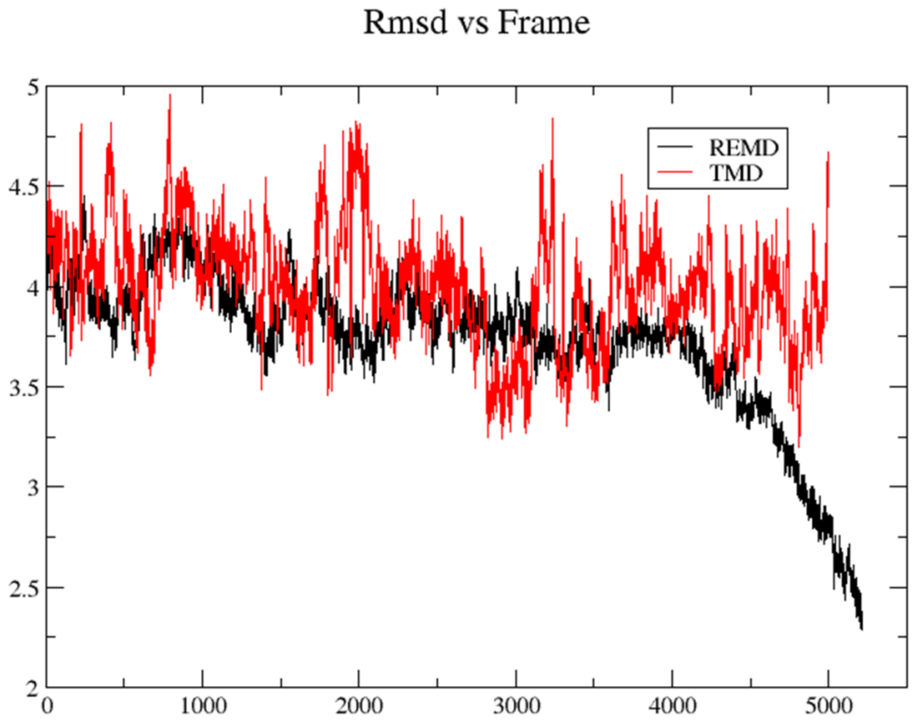

Supplementary Figure 8. Conformational switch between open and closed states. RMSD between open and closed states (regions: Glu99-Phe109, Thr159-Val164) in two MD techniques. TMD is more successful in obtaining the closed state from the open state as shown by their closing RMSD.

Supplementary Table 1. Statistics for data collection and refinement.

Data set	WT apo open form	WT apo closed form	H216A apo open form	H216A apo closed form	H247A apo open form	H247A tetra-tri peptide bound	H247A penta-peptide bound	WT apo closed form (Zn SAD)
A. Data collection								
X-ray source	PLS-5C	PLS-5C	PLS-5C	PLS-5C	PLS-7A	PLS-7A	PLS-7A	PLS-7A
X-ray wavelength (Å)	1.0000	1.0000	1.0000	1.0000	1.0000	1.0000	1.0000	1.2824
Space group	$P6_1$	$P3_221$	$P6_1$	$P2_1$	$P6_1$	$P6_1$	$P6_1$	$P3_221$
Unit cell length (a, b, c, Å)	114.5, 114.5, 57.9	57.9, 57.9, 152.7	113.2, 113.2, 54.5	82.3, 105.6, 86.2	114.3, 114.3, 55.6	114.8, 114.8, 55.6	114.3, 114.3, 55.9	58.1, 58.1, 152.5
Unit cell angle (α, β, γ, °)	90, 90, 120	90, 90, 120	90, 90, 120	90, 107.2, 90	90, 90, 120	90, 90, 120	90, 90, 120	90, 90, 120
Resolution range (Å)	50.0–1.86 (1.89–1.86)[a]	50.0–1.66 (1.69–1.66)[a]	50.0–2.10 (2.14–2.10)[a]	50.0–2.04 (2.08–2.04)[a]	50.0–1.92 (1.95–1.92)[a]	50.0–2.20 (2.24–2.20)[a]	50.0–2.40 (2.44–2.40)[a]	50.0–1.72 (1.75– 1.72)[a]
Total / unique reflections	804,553 / 36,515	481,855 / 35,949	501,309 / 23,195	622,169 / 89,706	619,581 / 31,366	425,878 / 22,451	62,308/ 16,610	1,141,822 / 32,441
Completeness (%)	100.0 (100.0)[a]	100.0 (100.0)[a]	99.8 (100.0)[a]	99.8 (99.1)[a]	98.7 (99.2)[a]	99.9 (100)[a]	99.0 (100)[a]	99.9 (100)[a]
Average I/σ (I)	78.3 (5.6)[a]	51.1 (4.9)[a]	75.2 (8.4)[a]	42.3 (5.2)[a]	73.7 (9.0)[a]	74.6 (14.4)[a]	23.8 (2.1)[a]	102.7 (11.4)[a]
R_{merge}[b] (%)	7.7 (90.0)[a]	6.2 (45.6)[a]	13.4 (84.9)[a]	10.4 (69.0)[a]	10.0 (93.4)[a]	12.3 (96.1)[a]	7.2 (79.4)[a]	12.0 (68.1)[a]
B. Model refinement statistics								
Resolution range (Å)	50.0–1.86	50.0–1.66	50.0–2.10	50.0–2.04	50.0–1.92	50.0–2.20	50.0–2.40	50.0–1.72
R_{work} / R_{free}[c] (%)	17.2 / 19.9	17.3 / 20.6	18.2 / 21.3	16.7 / 19.4	17.1 / 19.7	16.1 / 19.9	17.8 / 22.6	21.7 / 26.0
Monomers per asymmetric unit	1	1	1	3	1	1	1	1
Number of non-hydrogen atoms / average B-factor (Å ^{2})								
Protein	2,028/ 43.8	2,038 / 31.8	2,038 / 51.5	6,249 / 35.1	2,014 / 41.4	2,014 / 46.8	2,006 / 60.5	2,038 / 32.9
Water oxygen	242 / 50.3	210 / 41.8	136 / 52.0	651 / 44.2	135 / 47.3	157 / 54.6	99 / 60.8	218 / 41.8
Zn^{2+}	1 / 33.0	1 / 21.9	1 / 54.0	3 / 26.1	1 / 39.8	1 / 53.7	1 / 83.0	1 / 34.0
Tartrate	10 / 63.3	-	-	30 / 51.2				
Citrate	-	13 / 34.5						13 / 35.2
Glycerol	18 / 58.8	-						
Peptide					-	30 / 77.3	23 / 85.0	
Sulfate			5 / 72.2	-				

Supplementary Table 1. *contd.*

R.m.s. deviations from ideal geometry								
Bond lengths (Å) / bond angles (°)	0.007 / 0.80	0.006 / 0.78	0.008 / 0.89	0.007 / 0.84	0.006 / 0.79	0.007 / 0.91	0.008 / 1.09	0.006 / 0.79
Protein-geometry analysis								
Ramachandran favored (%)	96.5	95.3	92.7	95.5	93.3	94.9	95.6	95.3
Ramachandran allowed (%)	3.5	3.9	5.0	3.9	5.5	4.7	3.2	4.3
Ramachandran outliers (%)	0	0.8	2.3	0.6	1.2	0.4	1.2	0.4

Footnotes for Supplementary Table 1

[a]Values in parentheses refer to the highest resolution shell.

[b]$R_{merge} = \Sigma_{hkl}\Sigma_i \mid I_i(hkl) - \langle I(hkl)\rangle \mid / \Sigma_{hkl}\Sigma_i\ I_i(hkl)_i$, where $I(hkl)$ is the intensity of reflection *hkl*, Σ_{hkl} is the sum over all reflections, and Σ_i is the sum over i measurements of reflection *hkl*.

[c]$R = \Sigma_{hkl} \mid |F_{obs}| - |F_{calc}| \mid / \Sigma_{hkl} |F_{obs}|$, where R_{free} was calculated for a randomly chosen 5% of reflections, which were not used for structure refinement and R_{work} was calculated for the remaining.

Supplementary Table 2. Primers for structure, *C. jejuni* morphology, and invasion studies.

Primers	Sequence (5′-3′)
Pgp3-NdeI-F	GCGCTCATATGATGAAAGCCTTATGGCTTTTTTTAAG
Pgp3-XhoI-R	GCGCTCTCGAGTCATTGAAAAATAGCATTAAATTTGGAT
Pgp3-H216A-F	GAATTTATTCACAATATTATGCTCTTTCTAAAATCGATGTTAA
Pgp3-H216A-R	TTAACATCGATTTTAGAAAGAGCATAATATTGTGAATAAATTC
Pgp3-H247A-F	GTGGTAGGGTAAGTGGGCCGGCTTTGCATTTTGGAATTTTAGC
Pgp3-H247A-R	GCTAAAATTCCAAAATGCAAAGCCGGCCCACTTACCCTACCAC
Pgp3-PstI-F	TTTCTGCAGCCTAGCGAAGTAATCATCACTT
Pgp3-SalI-R	TTTGTCGACAACTTCTAGTCATTGATGAGGG
Pgp3-inverse-F	GCTGGAGGCAAACAAGTTGA
Pgp3-inverse-R	AGCTTGACCTTTGATGAGTTCT
Kan-F	GCGATGAAGTGCGTAAG
Kan-R	CGGCTCCGTCGATACTATG
Pgp3-comple-F	AAATCTAGACCGAACAAGCTCCACTTCCA
Pgp3-comple-R	AAATCTAGATGCTTGGCTAAAAGTGTG CC

Supplementary References

1. Jamieson, A. G. et al. Positional scanning for peptide secondary structure by systematic solid-phase synthesis of amino lactam peptides. *J. Am. Chem. Soc.* **131**, 7917-7927 (2009).
2. Pellicciari, R., Natalini, B. & Marinozzi, M. L-Vinylglycine from L-Homoserine. *Synth. Commun.* **18**, 1715-1721 (1988).
3. Roychowdhury, A. & Boons, G. J. The synthesis of diaminopimelic acid containing peptidoglycan fragments using metathesis cross coupling. *Tetrahedron Lett.* **46**, 1675-1678 (2005).
4. Rees, D. O. et al. Synthesis of [1,2-13C2, 15N]-L-homoserine and its incorporation by the PKS-NRPS system of fusarium moniliforme into the mycotoxin fusarin C. *Chembiochem.* **8**, 46-50 (2007).
5. Roychowdhury, A., Wolfert, M. A. & Boons, G. J. Synthesis and proinflammatory properties of muramyl tripeptides containing lysine and diaminopimeric acid moieties. *Chembiochem.* **6**, 2088-2097 (2005).
