# Supplementary material for: Peptidoglycan reshaping by a noncanonical peptidase for helical cell shape in Campylobacter jejuni
Source: Nat Commun. 2020 Jan 23;11:458. doi: 10.1038/s41467-019-13934-4 (PMC6978369; doi:10.1038/s41467-019-13934-4)
Supplement: Supplementary file 3 — Description of Additional Supplementary Files [file 41467_2019_13934_MOESM3_ESM.pdf]

Description of Additional Supplementary Files

File name: Supplementary Movie 1

Description: Supplementary Movie 1 is the molecular dynamics (MD) movie depicting conformational transition. Movie represents conformational transition between open and closed states.
